# The Impact of FDA-Approved Novel Agents for Steroid-Refractory Chronic Graft vs. Host Disease on Treatment Patterns and Outcomes—A Single-Center Longitudinal Cohort Analysis

**DOI:** 10.3390/cancers16203521

**Published:** 2024-10-17

**Authors:** Gil Fridberg, Odelia Amit, Chen Karni, Dina Tshernichovsky, David Shasha, Vanessa Rouach, David Varssano, Amir Bar-Shai, Ilan Goldberg, Gilad Wasserman, Irit Avivi, Ron Ram

**Affiliations:** 1 Bone Marrow Transplantation Unit, Tel Aviv Sourasky Medical Center, Tel Aviv 6423906, Israel; gilfr@tlvmc.gov.il (G.F.); odeliaa@tlvmc.gov.il (O.A.); chenk@tlvmc.gov.il (C.K.); dinat@tlvmc.gov.il (D.T.); iritavi@tlvmc.gov.il (I.A.); 2 Faculty of Medical and Health Sciences, Tel Aviv University, Tel Aviv 6997801, Israel; davidsha@tlvmc.gov.il (D.S.); vanessar@tlvmc.gov.il (V.R.); davidva@tlvmc.gov.il (D.V.); amirbs@tlvmc.gov.il (A.B.-S.); ilango@tlvmc.gov.il (I.G.); giladwas@tlvmc.gov.il (G.W.); 3 Infectious Disease Unit, Tel Aviv Sourasky Medical Center, Tel Aviv 6423906, Israel; 4 The Institute of Endocrinology, Tel Aviv Sourasky Medical Center, Tel Aviv 6423906, Israel; 5 Department of Ophthalmology, Tel Aviv Sourasky Medical Center, Tel Aviv 6423906, Israel; 6 The Institute of Pulmonary Medicine, Tel Aviv Sourasky Medical Center, Tel Aviv 6423906, Israel; 7 Department of Dermatology, Tel Aviv Sourasky Medical Center, Tel Aviv 6423906, Israel; 8 Department of Oral Medicine, Tel Aviv Sourasky Medical Center, Tel Aviv 6423906, Israel

**Keywords:** GVHD, allogeneic HCT, steroids, ruxolitinib, ibrutinib

## Abstract

**Objectives**—chronic graft vs. host disease (cGVHD) is associated with substantial morbidity and mortality. We aimed to analyze advances in treatment strategy and outcomes during the last decade due to the incorporation of novel immunosuppressive therapy (IST) drugs in the armamentarium. **Methods**—we retrospectively analyzed all patients > 18 years with cGVHD after their first hematopoietic cell transplantation (HCT) between 2012 and 2020 (n = 91), divided into three treatment periods: 2012–2014, 2015–2017, and 2018–2020 (groups 1, 2, and 3, respectively). **Results**—mean cumulative steroid dose and dose/total cGVHD-treatment days was lower in groups 2–3 compared to 1 (*p* = 0.008 and *p* = 0.042, respectively). The median IST-free survival was 79 (95%CI54–94) months, with more patients in group 3 (47% (95%CI 25–54%) discontinuing IST at 3 years, *p* = 0.1). Groups 2–3 compared to 1 had better glycemic control (*p* < 0.01), higher bone density (*p* = 0.06), and fewer cardiovascular events. The number of admissions/patient dropped from 0.7/year in group 1 to 0.24/year and 0.36/year in groups 2–3, respectively (*p* = 0.36). Employment reintegration was higher in groups 2–3 compared with 1 (*p* = 0.05) and so was earlier return to work (*p* = 0.01). There were no differences in survival outcomes. **Conclusions**—the incorporation of novel agents appears to be associated with reduced overall steroid burden, improved cGVHD control, and fewer long-term side effects.

## Simple Summary

Chronic graft vs. host disease (cGVHD) is a major complication that appears after allogeneic hematopoietic cell transplantation. As result, this has a significant and detrimental impact on both the quality of life and survival of patients after transplantation. In recent years, three novel therapies have been approved by the FDA for the management of cGVHD. To date, previous studies have not analyzed the effect of these novel drugs on other health-associated conditions such as metabolic syndrome, bone health, return to employment, mental health, and other para-medical outcomes. In this study, during three different time periods, we aimed to assess and highlight the association between novel advanced treatments for cGVHD and their impact on the quality of life of patients after allogeneic transplantation.

## 1. Introduction

Chronic graft vs. host disease (cGVHD), explicitly steroid-refractory disease, is associated with substantial morbidity and mortality in patients undergoing allogeneic hematopoietic cell transplantation (HCT) [1,2,3]. The one-year overall survival rate of patients with steroid-refractory cGVHD is approximately 80%, with continuous deterioration in survival among both responsive and non-responsive patients [4]. As a result, these patients suffer from its significant impacts on physical, social, economic, and psychological aspects of their lives, thus leading to a lower general quality of life. Until recently, treatment options for managing steroid-refractory chronic GVHD were both limited and had only a modest effect on the clinical manifestations of the disease. These options included extra-corporal photopheresis, mycophenolic acid, rituximab, and imatinib, as well as other strategies that were based on small studies, showing only mild efficacy. 

In the last 3 years, three novel drugs have been approved by the FDA for the treatment of chronic GVHD—ibrutinib, ruxolitinib, and belumosudil. These medications were approved based on studies showing improvement in symptom control with an acceptable toxicity profile, yet, to date, no survival benefit has been shown [4,5,6,7,8]. As opposed to steroids that suppress both the immune response and reduce inflammation in a non-specific fashion, these novel agents have specific targets that selectively inhibit canonical pathways associated with chronic GVHD [9]. Ruxolitinib is a selective JAK 1/2 inhibitor that modifies intracellular kinases of immune cells, including components of both the innate and adaptive immune system [10]. Ibrutinib is a selective inhibitor of Bruton’s tyrosine kinase, which inhibits signal transduction of the B-cell receptor [11], and belumosudil is a selective inhibitor of rho-associated coiled-coil-containing protein kinase-2, an important signaling pathway that regulates several subclasses of T cells, mainly the balance between Th17 and regulatory T-cells associated with profibrotic pathways [12].

While, in general, the safety profile of these novel FDA-approved medications appears to be better compared to prolonged steroid therapy, currently there is a lack of data regarding the impact of these novel agents on non-GVHD secondary outcomes [13]. Since 2018, in our center, access to novel immunosuppressive therapies (ISTs) for steroid-refractory GVHD has become available via clinical studies, early-access programs, and private insurances.

In this longitudinal, retrospective, single-center study, we aimed to analyze advances in the treatment pattern over the last decade and their impact on long-term follow-up endpoints in patients with steroid-refractory chronic GVHD.

## 2. Methods

### 2.1. Patients and Initial Treatment for Chronic GVHD

The electronic charts of all consecutive patients who underwent allogeneic HCT between January 2012 and December 2020 in the Tel Aviv Sourasky Medical Center were systematically reviewed. All patients signed informed consent for data reporting, and this study was approved by the local ethics committee.

Eligibility criteria for this analysis included adult patients (>18 years) with chronic GVHD graded according to the 2005 National Institutes of Health (NIH) diagnostic and scoring criteria [14,15], who were initially treated with systemic steroids. The date for the diagnosis of cGVHD was defined as the day systemic treatment for cGVHD was initiated. The severity score for involved organs, overall cGVHD severity, and the clinical phenotype was determined using the most recent NIH criteria [14,15]. In general, patients were given systemic steroids (prednisone) at a dose of 0.5–1 mg/kg as first-line treatment in cases of moderate–severe chronic GVHD or in cases of mild chronic GVHD with either progressive onset or with concomitant low platelet count at presentation [16,17]. Patients continued cyclosporine or restarted cyclosporine in cases where therapy was recently discontinued prior to chronic GVHD onset. Topical treatment was not recorded in this analysis. Steroid doses were recorded at every clinic follow-up. All of the patients were evaluated and treated by an experienced team consisting of 3 physicians and a designated transplant nurse in the long-term follow-up clinic in our center. Gynecology assessment was routinely performed from 3 months post transplant, documented and managed by a specialist. We divided the patient cohort into 3 treatment periods according to clinical trials available/FDA-approved drugs: 2012–2014 (Group 1), 2015–2017 (Group 2), and 2018–2020 (Group 3).

### 2.2. Second Line Treatment

Steroid-refractory disease or dependence was defined according to the NIH consensus criteria [15]. Responses were assessed weekly or fortnightly from the start of a new line of treatment until the stabilization of the condition and thereafter, according to physician discretion. Patients with steroid-refractory disease/dependence were eligible for second-line therapy, which included rituximab, imatinib, extra-corporal photopheresis (ECP), and sirolimus. In recent years, ruxolitinib, ibrutinib, and belumosudil have become available.

### 2.3. Evaluation and Definition of Outcomes

Surveillance for metabolic conditions was performed according to a local preplanned protocol that included screening of several domains. Patients had serial blood tests for HBA1C and lipid profiles from 3 months after HCT and at 1 year after HCT and thereafter, annually. All patients were referred for bone density analysis at 1 year after HCT and thereafter as applicable. Cardiovascular events included myocardial infarction, ischemic heart disease onset, and cerebral vascular accidents. A return-to-work consultation was performed from 3 to 6 months after HCT and was based on local guidelines. The annual number of admissions to the hospital was calculated from the onset of chronic GVHD to the last follow-up. Depression and/or anxiety was defined according to documented medical treatment or during psychological follow-up and/or therapy.

### 2.4. Supportive Care

GVHD prophylaxis, in the majority of the patients, included cyclosporine and methotrexate with the addition of ATG in patients receiving allografts from unrelated donors and, in the case of sibling allografts, male recipients from female donors. All patients received prophylaxis against pneumocystis jiroveci, as well as for herpes simplex and varicella zoster virus.

All patients received antifungal prophylaxis with posaconazole during steroid use above the dose of 0.5 mg/kg. Patients were not required to continue prophylaxis once the dose was reduced below 0.5 mg/kg, irrespective of concomitant administration of other immunosuppressive medications. Immunoglobulins were not routinely used but were administered if IgG levels were below 3.5 gr/L.

### 2.5. Statistical Analysis

Data were analyzed as of June 2022. The baseline and treatment characteristics are described using median and range for continuous variables and number and percentages for categorical variables. To compare steroid burden while overcoming inherent confounders between the 3 groups, we calculated the mean cumulative dose of steroid exposure for each patient as mg/kg/day (prednisone equivalent) and the ratio between the cumulative dose and the total chronic GVHD treatment days. IST-free, non-relapse mortality and overall survival were estimated using the Kaplan–Meier method, with death treated as a competing risk in the analyses of IST-free survival and relapse as a competing risk in the analysis of non-relapse mortality. The Log-rank method was used to compare among the 3 groups. Cox regression was used to identify risk factors for overall survival. For each analysis, hazard ratios (HRs) and 95% confidence intervals (95% CIs) are given together with *p* values for comparisons with the reference category. All *p* values were derived from likelihood ratio statistics and are two sided. Data were analyzed using the SPSS version 29.0.

## 3. Results

### 3.1. Patient and Chronic GVHD Characteristics

**Figure 1:**
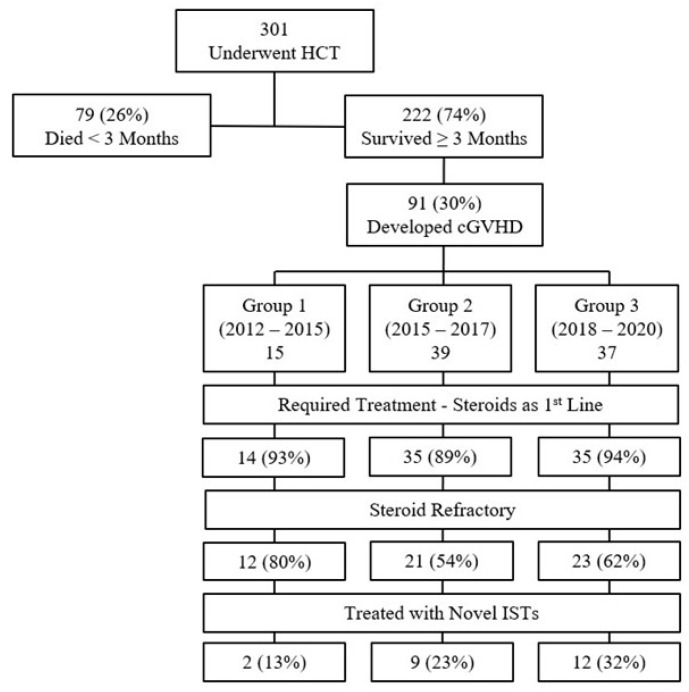
Patient flow chart.

A patient flow chart is presented in Figure 1. Of the 301 patients that underwent allogeneic HCT between 2012 and 2020, 222 patients survived ≥3 months and were assessed for chronic GVHD, and 91 patients (41%) met the inclusion criteria—15 patients in Group 1 (2012–2014), 39 patients in Group 2 (2015–2017), and 37 patients in Group 3 (2018–2020). Patients’ characteristics by time periods are presented in Table 1. The most common indication for HCT was AML. The myeloablative preparative regimen was more commonly administered in Group 1, comparing to Groups 2 and 3 (87% vs. 62% and 41%, respectively); however, the percentage of patients receiving a TBI-containing regimen were similar between the three groups. In addition, there was an increased percentage of older patients and URD grafts in Groups 2 and 3, compared to Group 1. Manifestations of chronic GVHD were balanced across groups, with the skin and mouth being the most common organs affected.

### 3.2. Steroid Burden and Subsequent Treatment Patterns

**Figure 2:**
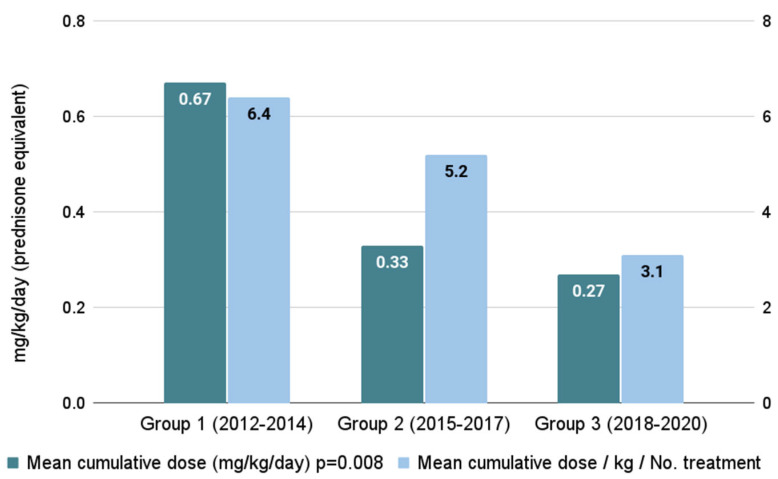
Accumulated steroid burden across time periods.

The mean cumulative steroid dose was significantly lower in Groups 2 and 3 (0.33 ± 0.24 mg/kg/day and 0.27 ± 0.3 mg/kg/day, respectively) compared to Group 1 (0.67 ± 0.79 mg/kg/day), *p* = 0.008, Figure 2. Similarly, the proportion of cumulative steroid dose to total cGVHD treatment days was lower in Groups 2 and 3 compared to Group 1 (5.2 ± 4, 3.1 ± 1.9 and 6.4 ± 8.9, respectively; *p* = 0.042).

Steroid therapy as first-line treatment was found in 92% of patients (the remaining did not require systemic therapy). Their use in second-line treatment was seen in 12 (86%), 21 (60%), and 23 (66%) patients in Groups 1, 2, and 3, respectively. Second-line treatment included ECP (n = 10, 16%), non-ECP and non-novel agents (n = 30, 47%), and novel agents (n = 14, 22%). The most common second-line therapy was tacrolimus (25%), ECP (28%), and mycophenolate mofetil (39%) in Groups 1, 2, and 3, respectively. Ruxolitinib was given to 26 patients (28%) from the entire cohort as second-line treatment (n = 13, 14%), third-line treatment (n = 5, 5%), or >3 line (n = 8, 9%), and ibrutinib was given to 6 patients as a third-line therapy (n = 6, 7%). Second-line novel agents were given to zero (0%), four (20%), and nine (39%) patients in Groups 1, 2, and 3, respectively. ECP was utilized for two (17%), six (30%), and two (9%) patients, respectively.

There was no difference between the three groups in the median time from first- to second-line treatment; however, Group 3 showed the shortest median time at 3.3 months (IQR 1.3–16.5, (*p* = 0.07). A landmark analysis showed that incidences of 6-month, second-line, adjusted, failure-free survival were 66% (95%CI 44–88%), 56% (95%CI 45–67%), and 76% (95%CI 66–86%), respectively; *p* = 0.7. The 2-year cumulative percentage of patients who required third-line treatment was higher in Groups 1 and 2 (68% and 57%) compared to Group 3 (45%); *p* = 0.08.

### 3.3. Long-Term Outcomes

Table 2 depicts the long-term outcomes of the three groups. A higher number of patients in Group 1 had osteopenia and osteoporosis diagnosed via bone density scan compared to patients in Groups 2 or 3 (n = 8, 62% vs. n = 12, 33% and n = 5, 16%, respectively, *p* = 0.06). Patients in Groups 2 and 3 showed better glycemic control (maximal HbA1C levels of 6.5 mg/dL compared to 5.9 and 5.7, respectively; *p* < 0.01) and a non-statistically significant lower incidence of cardiovascular events, as well as a mean number of hospitalizations/patient (n = 0.7/year vs. n = 0.24/year and n = 0.36/year, respectively).

There was a lower incidence of employment reintegration among patients in Group 1 compared to patients in Groups 2 and 3 (20% vs. 56% and 43%, respectively; *p* = 0.05), and reintegration occurred more rapidly with mean months of 29 (±9.8), 11.9 (±4.3), and 9.5 (±3.8), respectively (*p* = 0.01). There was no difference between the groups in the rates of diagnosed depression and treatment (*p* = 0.58).

**Figure 3:**
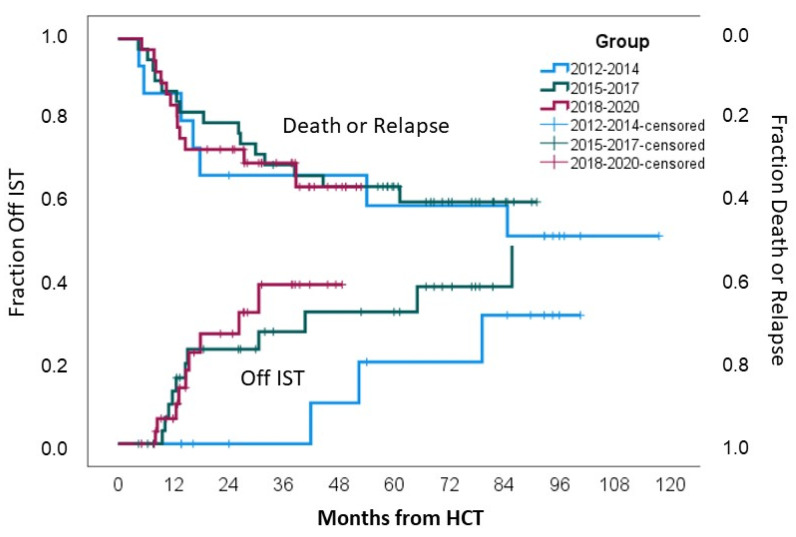
Mortality or relapse rates and IST discontinuation.

Incidences of IST-free survival for the whole cohort at 24, 36, 48, and 60 months were 27% (95%CI 15–39%), 37% (95%CI 23–51%), 38% (95%CI 18–58%), and 42% (95%CI 21–63%), respectively. At 24 months, 0% of the patients in Group 1 were IST-free, while in Groups 2 and 3, 31% and 36% of the patients, respectively, were IST-free; *p* = 0.1, Figure 3.

There was no difference in 3-year relapse incidence (*p* = 0.54), non-relapse mortality (*p* = 0.69), and overall survival (0.68) between the three groups, as indicated in Table 2 and Figure 3.

Cox regression analysis identified a lower platelet count at the onset of cGVHD and the employment of a reduced intensity regimen as being associated with shorter survival rates (HR-1.1, *p* = 0.005, and HR-3.5, *p* = 0.049, respectively), while the patient’s group (1–3), sex, age, and eosinophil count did not impact survival.

## 4. Discussion

In this longitudinal retrospective analysis, we focused on several cGVHD-associated bio-psychosocial endpoints that have not been substantially investigated in the recent era of novel agents. The evolution in the last decade at our center to incorporate novel agents in early lines of therapy showed several changes in patterns of outcomes. We showed that cumulative steroid dose was lower, and the success of second-line treatment was higher in patients treated in recent years. These trends resulted in fewer metabolic complications and faster employment reintegration. Due to the relatively short follow-up period, we did not demonstrate the survival benefit for the above-mentioned trends.

The characteristics of the patients in the three cohorts were found to be similar to patterns seen in other transplantation centers across Europe and the US, with a gradual increase in the age of the recipient that results in a decrease in the percentage of patients given myeloablative preparative regimens [18]. While myeloablative regimens (compared to those of reduced intensity) are associated with greater epithelial and endothelial damage that may subsequently result in the discharge of cytokines and initiation of acute GVHD, chronic GVHD is mainly characterized by an impairment of immunologic tolerance that interferes with both innate and adoptive immunity [2,18]. As such, there are conflicting data regarding the effect of the intensity of conditioning on the incidence of GVHD, and only TBI-based regimens have been consistently shown to impact the incidence of chronic GVHD [3]. In our cohort, while the overall percentage of patients receiving myeloablative regimens decreased in recent years, the percentage of patients receiving TBI-containing regimens were similar (22–27%).

First-line treatment of moderate–severe cGVHD with steroids is considered the standard practice in many centers; however, this treatment results in substantial morbidity [19,20]. We observed shorter time to second-line treatment in Group 3. This probably stemmed from the increased availability of novel medications included within the standard health insurance package. In our study, the steroid cumulative burden dropped from 0.67 mg/kg/day to 0.27 mg/kg/day (59% reduction), and the cumulative steroid dose to total cGVHD treatment days dropped from 6.4 to 3.1 once novel agents were incorporated, as was previously shown by others [5,21,22].

Our findings suggest that the metabolic profiles of steroid-refractory cGHVD patients could be improved once novel agents are available. This was reflected by better glycemic control (a decline in HbA1C from 6.5 mg/dL to 5.7 mg/dL), bone density (a similar percentage of osteopenia with a decline in overt osteoporosis from 62% to 16%), and reduced cardiovascular morbidity (the latter not statistically significant) in routine, timed follow-up assessments from Groups 1 to 2 and 3. All domains are well known to be affected by chronic steroid use and by the continuation of other IST drugs, as well as uncontrolled cGHVD, thus providing evidence for a clinically measurable benefit in a real-life setting [23,24]. As described in previous studies, it is challenging to establish a definitive association between steroid use and metabolic outcomes since the vast majority of patients in these studies are treated with steroids; thus, cohorts comprising homogenously exposed patients are evaluated [25]. In our cohort, the different exposure rates to steroids between the groups, as reflected by mean cumulative steroid dose, were able to provide insight into this association. It is important, however, to note that over the assessed time period, the LT follow-up clinic evolved to include the early introduction of dietician and endocrinology consultations. In addition, we introduced early vitamin D/calcium screening, as well as lipid and metabolic profile testing, leading to earlier diagnosis and intervention. This highlights the importance of combining appropriate medical therapy with risk reduction and intervention.

Of note, with lower steroid cumulative dose consumption and with better GVHD control, we showed that there was a 66% reduction in all admission rates from 0.7 to 0.24 admissions/year/patient.

Employment reintegration can be considered a global marker for symptom control, the complication of ISTs, and quality of life in cGVHD patients. Yu et al. recently reported that among patients experiencing chronic GVHD 71.3% supposed that GVHD-related symptoms were associated with a loss of income [19]. We observed higher, as well as faster, return-to-work rates, emphasizing what seems to be a rapid benefit associated with novel IST agents. As previously mentioned, during these years, the long-term follow-up clinic introduced the addition of physiotherapy review, long-term follow-up, and home physiotherapy early on to maintain bone strength and range of movement. This may contribute to earlier return-to-work rates due to the better side-effect profiles of novel ISTs (for example, the lack of steroid-induced myopathy), thus allowing for greater benefits from physical therapy to be derived.

Although not reaching statistical significance, when novel agents were not available, patients had higher rates of depression or anxiety. This domain requires further research to decipher its cause and effect and to determine whether these psychological issues result from poor symptom control of cGVHD, side-effect profiles of older IST therapies, unemployment rates, or a mixture of cofounding factors. Considering this, in our LT follow-up clinic, we introduced support groups for patients to support long-term mental health, potentially contributing to the lack of statistical significance regarding anxiety and depression diagnosis.

Finally, with regard to genital GVHD, throughout the study period there was a greater awareness among physicians of this issue. As such, patients were routinely assessed in a specialty clinic, and topical therapy was introduced early. This pre-emptive approach may lower the rates of genital GVHD that require systemic therapy or an interventional approach.

Several studies have evaluated disability associated with chronic GVHD but have mainly assessed this outcome based on physician-assessed clinical manifestation scoring (Flowers criteria) [26,27]. Others focused on patients who reported outcomes of disability and who returned to work and found that 2/3 of the patients with chronic GVHD reported severe physical and work-related disabilities [28]. To our knowledge, this is the first study attempting to analyze the impact of treatment with novel agents on these domains and in addition to other metabolic and clinically relevant complications associated with GVHD. In line with prior results from clinical trials, the observed changes in treatment patterns and secondary outcomes in our study did not translate into survival benefit [5,15]. Nevertheless, we did not observe differences in the relapse rate between the three groups, with a 20% relapse incidence, similar to the percentage previously reported [29].

Our study is limited by several factors. First, this is a single-center retrospective study and contains respective associated biases, with difficulties in attributing changes in treatment patterns, and consequently secondary outcomes, to a single factor. Nevertheless, the balanced patients’ characteristics across groups, the consistency in the clinical evaluation, and the therapeutic decision-making process resulting from all patients being managed by same three physicians in a single center did minimize potential confounders. Second, all three groups had a relatively small number of patients, and the sample size was too small to draw long-term conclusions. Larger prospective trials with a longer follow-up period, focusing on the quality of life within multiple domains, may further solidify our results and clinical implications.

In conclusion, in this longitudinal, retrospective, single-center study, we showed that, once becoming available, novel agents can change treatment patterns, which can consequently translate to superior secondary clinical and quality-of-life outcomes in cGHVD patients. This study also highlights the importance of regular long-term follow-up surveillance to better control long-term-transplantation-associated toxicities and improve overall quality of life.

## 5. Conclusions

Chronic GVHD is a common and morbid complication of allogeneic HCT. After many years of lacking effective therapies in the field, three new therapies have been approved for patients who do not respond to first-line steroid therapy. We showed that these novel FDA-approved medications were associated with fewer metabolic complications, higher incidences of patients returning to work in a shorter time after transplant, lower rates of readmissions to the hospital, and better control of transplant-associated bone disease. While these outcomes are associated with the improvement of quality of life for patients, there was no difference in non-relapse mortality and no improvement in overall survival. A longer follow-up period and a well-designed prospective trial with a thorough evaluation of secondary outcomes may shed more light on the impact of these domains and thus contribute to further improvement in patients’ outcomes. We hypothesize that the use of novel IST agents with a safer side-effect profile and greater LT follow-up and intervention can lead to better quality of life for these patients, which may ultimately result in improved overall survival.

## Figures and Tables

**Table 1:** Baseline patients’ characteristics.

Domain		All Cohort 2012–2020 (n = 91)	Group 1 2012–2014 (n = 15)	Group 2 2015–2017 (n = 39)	Group 3 2018–2020 (n = 37)
Age (mean, ±SD)		50 ± 15.5	43 ± 15.6	45 ± 14.2	57 ± 14.1
Sex (Female, %)		39, 43%	8, 53%	18, 46%	13, 35%
Disease (N, %)	AML	48, 53%	6, 40%	22, 56%	20, 54%
	ALL	16, 18%	5, 33%	8, 21%	3, 8%
	Lymphoma	10, 10%	0, 0%	5, 13%	5, 14%
	MDS	8, 9%	2, 13%	2, 5%	4, 11%
	Other	9, 10%	2, 13%	2, 5%	5, 14%
Conditioning, myeloablative (N, %)		52, 57%	13, 87%	24, 62%	15, 41%
Donor (N, %)	URD, 10/10	53, 58%	5, 33%	22, 56%	25, 68%
	URD, 9/10	3, 3%	3, 20%	1, 3%	0
	Matched sibling	33, 37%	7, 47%	15, 39%	11, 30%
	Haploidentical	1, 1%	0	0	1, 1%
	Cord blood	1, 1%	0	1, 3%	0
	Female to male	19, 21%	3, 20%	11, 28%	8, 22%
cGVHD characteristics (N, %)	De novo	59, 65%	12, 80%	27, 69%	20, 54%
	Quiescent	12, 13%	1, 7%	4, 10%	7, 19%
	Progressive	20, 22%	2, 13%	8, 20%	10, 27%
cGVHD Grading (N, %)	Mild	14, 15%	1, 7%	8, 20%	5, 13%
	Moderate	32, 35%	4, 27%	11, 28%	17, 46%
	Severe	45, 49%	10, 67%	20, 51%	15, 40%
ECOG 0-1 (N, %)		74, 81%	9, 60%	29, 74%	36, 97%
Organ Involvement (N, %)	Skin	76, 84%	15, 100%	33, 85%	28, 76%
	Mouth	78, 86%	13, 87%	37, 94%	28, 76%
	Eyes	64, 70%	13, 87%	30, 77%	21, 57%
	Gastrointestinal	38, 42%	9, 60%	12, 31%	17, 46%
	Liver	41, 45%	7, 47%	17, 44%	17, 46%
	Lungs	26, 29%	7, 47%	11, 28%	8, 22%
	Joints/Fascia	39, 43%	9, 60%	16, 41%	14, 38%
	Genital (female)	20, 22%	5, 33%	11, 28%	4, 11%
	Other	3, 3%	0	2, 5%	1, 3%
	Eosinophilia	9, 10%	1, 7%	3, 8%	5, 14%
	Thrombocytopenia	29, 32%	6, 40%	7, 18%	16, 43%

URD—Unrelated donor, ECOG—Eastern Cooperative Oncology Group, AML—Acute Myeloid Leukemia, ALL—Acute Lymphoblastic Leukemia, MDS—Myelodysplastic Syndrome.

**Table 2:** Treatment patterns, Metabolic and Survival Outcomes.

Domain	Group 1 2012–2014 (n = 15)	Group 2 2015–2017 (n = 39)	Group 3 2018–2020 (n = 37)	*p*-Value
Steroid-refractory	12, 80%	21, 54%	23, 62%	0.21
Novel agent				
Second line	0	4, 10%	9, 24%	0.14
Second/third line	2, 13%	9, 23%	12, 32%	0.31
Median months to second line ^◊^ (IQR)	4.3 (1.7–11.2)	8.2 (2.9–20.1)	3.3 (1.3–16.5)	0.07
Discontinuation of IST at 36 months (CI)	20% (0–45%)	34% (21–47%)	47% (25–54%)	0.11
Maximal HbA1C (mg/dL)	6.5 ± 0.83	5.9 ± 0.69	5.7 ± 0.85	<0.01
CVA—n, %	1, 7%	1, 3%	1, 3%	0.73
MI/onset of IHD—n, %	4, 26%	4, 10%	3, 8%	0.16
Bone density ^¶^—n, %				0.06
Osteopenia	4, 31%	14, 39%	12, 38%	
Osteoporosis	8, 62%	12, 33%	5, 16%	
Number of admissions\year	0.7	0.24	0.36	<0.001
Return to work				
N patients, %	3, 20%	22, 56%	16, 43%	0.05
Months from HCT (mean)	29 ± 9.8	11.9 ± 4.3	9.5 ± 3.8	0.01
Depression/anxiety—n, %	6, 53%	14, 40%	10, 27%	0.58
3-year relapse incidence (CI) ^◊^	14% (5–19%)	12% (6–18%)	25% (8–42%)	0.54
3-year NRM (95%CI) ^◊^	14% (3–25%)	17% (4–30%)	26% (8–42%)	0.69
3-year OS (95%CI) *	66% (56–76%)	74% (60–88%)	63% (41–85%)	0.68

* Only among patients who received more than 1st line, ¶ Test performed 12–18 months from HCT, ◊ from onset of chronic GVHD. CI—Confidence Interval, CVA—Cerebrovascular Accident, IHD—Ischemic Heart Disease, IST—Immunosuppressive Therapy, MI—Myocardial Infarction, NRM—Non-Relapse Mortality, OS—Overall Survival.

## Data Availability

The data presented in this study are available on request from the corresponding author due to ethical reasons.
